# Effective radiation dose of a MSCT, two CBCT and one conventional radiography device in the ankle region

**DOI:** 10.1186/s13047-015-0067-8

**Published:** 2015-03-12

**Authors:** Juha Koivisto, Timo Kiljunen, Nils Kadesjö, Xie-Qi Shi, Jan Wolff

**Affiliations:** Department of Physics, University of Helsinki, Helsinki, Finland; Docrates Cancer Center, Helsinki, Finland; Image and Functional Odontology, Department of Dental Medicine, Karolinska Institutet, Huddinge, Sweden; VU University Medical Center, Amsterdam, The Netherlands

**Keywords:** X-ray imaging, CBCT, Organ dose, Effective dose

## Abstract

**Background:**

The aim of this study was to assess and compare the effective doses (ICRP 103) in the ankle region of X-ray imaging resulting from a multi slice computed tomography (MSCT) device, two cone beam CT (CBCT) devices and one conventional x-ray device.

**Methods:**

Organ dose measurements were performed using 20 metal oxide field effect transistor (MOSFET) dosimeters that were placed in a custom made anthropomorphic RANDO ankle phantom. The following scanners were assessed in this study: Siemens Sensation Open 24-slice MSCT-scanner (120 kVp, 54 mAs), NewTom 5G CBCT scanner (110 kVp, 2.3 - 59 mAs), Planmed Verity CBCT-scanner (90 kVp, 48 mAs), Shimadzu FH-21 HR direct radiography equipment (AP + LAT), (57 kVp, 16 mAs).

**Results:**

Measurements of the MSCT device resulted in 21.4 μSv effective dose. The effective doses of CBCTs were between 1.9 μSv and 14.3 μSv for NewTom 5G and 6.0 μSv for Planmed Verity. Effective doses for the Shimadzu FH-21 HR conventional radiography were 1.0 μSv (LAT) and 0.5 μSv (AP), respectively.

**Conclusions:**

Compared with a conventional 2D radiographic device, this study showed a 14-fold effective dose for standard MSCT and 1.3 -10 fold effective dose for standard CBCT protocols. CBCT devices offers a 3D view of ankle imaging and exhibited lower effective doses compared with MSCT.

## Background

Fractures of the foot are very common and account for approximately 10% of all fractures inflicted on the body [1]. Common causes of fractures of the foot are falling from heights and vehicle accidents [2]. The most commonly fractured foot bone is the calcaneus and accounts for approximately 60% of all foot fractures [3]. Talus fractures are the second most common fractures in the foot area and occur in 3-6% of cases [4]. To date, conventional radiographs have played a key role in the primary assessment of such fractures and provide quick, cheap and low-dose images. However, in complex fracture cases, conventional radiographs have limitations in their dynamic range and image contrast. Furthermore in conventional radiographs the object of interest can be obscured by overlying structures [5].

In many hospitals, multi-slice computed tomography (MSCT) technology has completely replaced conventional radiography providing all the necessary information in one examination that formerly required multiple studies [6]. The great benefits of 3D imaging devices are, however, often associated with a disproportional increase in radiation dose, an increase in part attributed to overutilization [7] without taking the ALARA (As low as reasonable achievable) principle into account [8].

One method of minimizing radiation doses in 3D imaging of the foot and ankle is to implement cone-beam computed tomography (CBCT) technology. CBCT technology has only recently been applied for the imaging of extremities such as the foot, ankle, knee, wrist and shoulder, and offers high spatial resolution, easy installation and low radiation dose [9-11] at a lower cost when compared with conventional CT modalities [12]. The novel CBCT devices offer the possibility of imaging the lower extremities under weight-bearing conditions (patient in standing position) and subsequently offers new possibilities to study osseous changes, for instance, degenerative joint disease of the knee, ankle and foot [13]. Both MSCT and CBCT are known to produce higher levels of radiation when compared with the conventional X-ray devices that were previously used. This has aroused a growing interest in effective dose measurements that have been commonly conducted using the CT dose index (CTDI) method [14,15] or by using optically stimulated luminescence (OSL) dosimeters [9]. The aim of this study was to assess the organ and effective doses International Commission on Radiological Protection (ICRP 103) [16] in the ankle area for two newly introduced CBCT devices, and to compare the corresponding MSCT and conventional radiography doses using metal oxide field effect transistor (MOSFET) dosimeters.

## Methods

### X-ray devices

**Figure 1:**
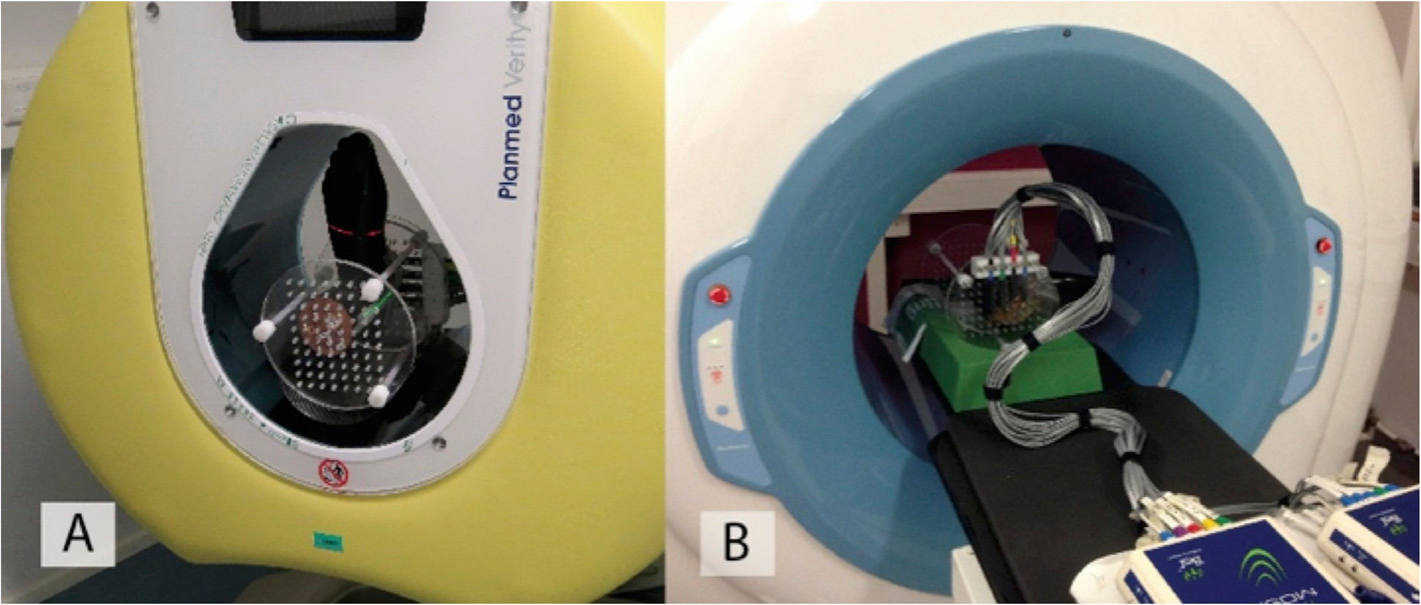
**The measurement setup for the Planmed Verity CBCT scanner (A) and the NewTom 5G CBCT scanner (B).**

Effective doses were calculated form organ doses obtained on one MSCT device, two CBCT devices and one conventional radiographic device: Siemens Sensation Open 24-slice scanner (Siemens, Forchheim, Germany), Planmed Verity CBCT-scanner (Figure 1A) (Planmed Oy, Helsinki, Finland), NewTom 5G CBCT scanner (Figure 1B) (NewTom 5G®, QR, Verona, Italy) and Shimadzu FH-21 HR direct digital radiography equipment (Shimadzu Corporation, Kyoto, Japan).

In order to attain comparative measurements, the MSCT scan length and the conventional X-ray field size were matched to the NewTom 5G (180 mm) and Planmed Verity (160 mm) scan lengths. In addition the “12 × 8 cm” FOV was measured using the HiRes modality and the “15 × 12 cm” FOV was measured using the standard scan modality. The small “12 × 8 cm” FOV using “HiRes” scan of NewTom 5G was measured as it provided comparable mAs values to MDCT and Planmed Verity CBCT device.

Since ankle fractures are typically imaged using anterior-posterior (AP) and lateral (LAT) radiographic views, the sum doses obtained using both projections were used as a reference in the dose comparison. All measurements were performed using manufacturer recommended ankle examination mode and exposure parameters (Table 1). The NewTom 5G CBCT scanner, however, employs automatic exposure control where the mA is set based on patient size estimated from two scout images. Here the mA cannot be changed manually.

**Table 1:** **Exposure parameters of the CBCT and MSCT scanners and conventional X-ray devices**

	**Siemens**	**NewTom 5G**			**Planmed**	**Shimadzu**
	**Somatom**	**CBCT**			**Planmed**	**FH-21 HR**
	**Definition AS+**	**Hi Res**	**Standard**	**Standard**	**Verity**	**AP + LAT**
	**MSCT**	**“12 × 8”**	**“15 × 12”**	**“18 × 16”**	**CBCT**	**Radiography**
Potential (kV)	120	110	110	110	90	57
Tube current (mA)‡	54	11	1,5	0,6	8	-
Exposure time (s)	1.0	5,4	3,6	3,6	6	-
Q (mAs)	54	59	5,3	2,3	48	16
Slice thickness (mm)	0.6	-	-	-	-	-
Pitch (mm)	0.5	-	-	-	-	-
Voxel H (mm)	-	0.15	0.3	0.3	0.2	-
Voxel L (mm)	-	0.15	0.3	0.3	0.2	-
Voxel W (mm)	-	0.15	0.3	0.3	0.2	-
Scan angle	360°	360°	360°	360°	210°	-
Frame number	-	360	360	360	300	-
Scan FOV (mm)†	130 × 160	120 × 80	150 × 120	180 × 160	130 × 160	130 × 160

†For MSCTs and CBCT: diameter × length, for conventional X-ray width × height.

‡For NewTom 5G CBCT: mA is automatically adjusted using the “SafeBeam™” acquisition option.

### Phantom

**Figure 2:**
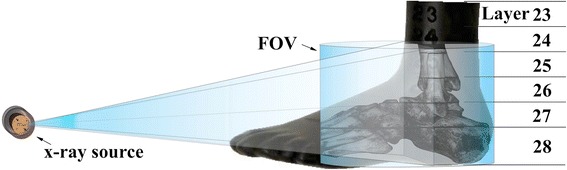
**The x-ray source, 3D FOV and ankle layers of the anthropomorphic RANDO phantom used in this study.**

All dose measurements were performed on a custom made anthropomorphic RANDO leg phantom (Radiation Analogue Dosimetry System; The Phantom Laboratory, Salem, NY, USA). The phantom comprised human leg bones molded in a soft tissue-equivalent material to match the attenuation and scattering properties of the bone and soft tissues of the human leg. The ankle area consisted of six layers numbered from 23 to 28 (Figure 2). Predrilled holes for the MOSFET dosimeters in each layer were arranged in a 1.5 cm × 1.5 cm matrix. The holes were delivered with factory fitted soft tissue equivalent plugs that were subsequently removed and replaced with MOSFET dosimeters. All ankle organ dose measurements were performed by directing the beam to layers 24–28 in the RANDO phantom (Figure 2). The phantom leg was positioned vertically in the MDCT and Planmed Verity CBCT device. However due to a different ankle support configuration and lack of space for the dosimeter cables on the NewTom 5G device a horizontal phantom position was used. This difference in positioning did however not affect the effective dose measurement due to the 360° gantry scan angle used on the NewTom 5G device. In total, between four to ten exposures were performed in the ankle area for each device, depending on the mAs of the device used, and the sum of all exposures was averaged.

### MOSFET dosimeters

All organ dose measurements in the ankle and foot region were performed using the same MOSFET dosimeters that were connected to a mobile TN-RD-70-W20 MOSFET device. The device comprised one TN-RD-38 wireless Bluetooth transceiver, four TN-RD-16 reader modules, twenty high-sensitivity TN-1002RD-H detectors and TN-RD-75 M software (Best Medical, Canada). The dosimeters can be used in either a high or low bias voltage providing high or low sensitivity response, respectively. In this study, all measurements were performed using the high bias voltage setting to achieve the best accuracy. Prior to the measurements, the mobile MOSFET device was calibrated using a RADCAL 1015 dosimeter and a RADCAL 10X5-6 ionization chamber (Radcal Corporation, Monrovia, CA, USA) that were referenced to the secondary standard dosimetry laboratory (SSDL) at the Finnish Radiation and Nuclear Safety Authority (STUK) that is traceable to primary standard dosimetry laboratory (PSDL). The dosimeter calibration procedure and angular sensitivity divergences and their implications were taken into consideration based on the results of an earlier study by Koivisto et al. [17]. Furthermore, the energy dependence of MOSFET dosimeters was considered to be negligible based on a study by Bower et al. [18].

**Figure 3:**
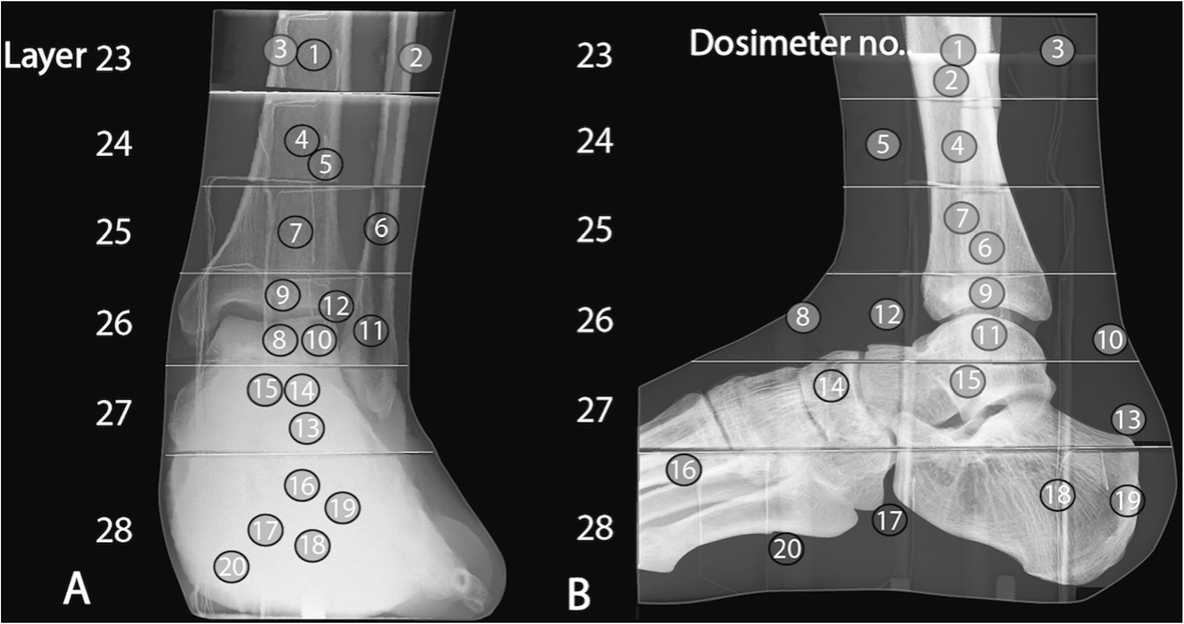
**The placement of MOSFET dosimeters in the leg phantom (AP (A), LAT B).**

The twenty MOSFET dosimeters (Figure 3, Table 2) were meticulously placed into layers 23–28 of the phantom to cover the most radiosensitive organs such as bone marrow and remainder tissues [16]. Since the largest contribution to the effective dose in the ankle area is caused by the bone marrow, one dosimeter was placed in the marrow of each layer to provide accurate bone marrow dose detection. Nine bone marrow dosimeters were placed inside the tibia, fibula, navicular, talus and calcaneus. Furthermore, four dosimeters were placed in the muscle area, two dosimeters in the lymphatic nodes, two dosimeters in the skin and two on the bone surface. The method presented in this study takes into account various depths within the phantom and the organ point doses on the skin (Figure 3). This was done to overcome the problems related to the non-uniform dose profiles produced by the MSCT and CBCT devices. MOSFET dosimeters were chosen for this study as they provide a nearly real-time dose assessment and have been found appropriate for extremity dose assessment in a previous study [17] and to provide TLD and Monte Carlo simulation comparable results according to earlier studies [19,20].

**Table 2:** **MOSFET dosimeter location in RANDO leg phantom**

**Dosimeter**			
**no.**	***Layer***	***Location***	**Tissue**
1	23	Tibia	Bone Marrow
2	23	Peroneus brevis	Muscle
3	23	Gastrocnemius	Muscle
4	24	Tibia	Bone Marrow
5	24	Tibialis anterior	Muscle
6	25	Fibula	Bone marrow
7	25	Tibia	Bone marrow
8	26	Superior extensor Retinaculum	Skin
9	26	Tibia	Bone marrow
10	26	Calcaneal tendon	Skin
11	26	Fibula	Bone marrow
12	26	Lymp.vein	Lymphatic nodes
13	27	Posterior calcaneal tuberosity	Bone surface
14	27	Navicular	Bone marrow
15	27	Talus	Bone marow
16	28	Metatarsals	Bone marrow
17	28	Lymp.vein	Lymphatic nodes
18	28	Calcaneus	Bone marrow
19	28	Calcaneal tuberosity	Bone surface
20	28	Flexor digitorum brevis	Muscle

The equivalent dose or radiation-weighted dose H_T_ for all organs or tissues T was calculated using the following equation [21,22]:

1 $$ {H}_T={w}_R{\displaystyle \sum_ifi\cdot {D}_{Ti}} $$

where the radiation weighing factor w_R_ = 1^**.**^ (Sv/Gy) for x-rays, *f*_*i*_ the mass fraction of tissue T in layer i, and D_*Ti*_ being the average absorbed dose of tissue T in layer i.

In this study, the term fraction irradiated (f_i_) used below describes the exposed and dosimetrically evaluated coverage of each studied organ in relative scale (Table 3). The studied organs were exposed to direct or scattered radiation during the scans.

**Table 3:** **ICRP 103 tissue weighting factors (wT), fractions irradiated (fi) and dosimeters used to calculate effective dose**

			***Dosimeter***
**Organ**	**wT**	**fi**	***number***
*Bone marrow*	0.12		
Tibia		0.007	1, 4, 7, 9
Fibula		0.001	6, 11
Navicular		0.001	14
Metatarsals		0.001	16
Calcaneus		0.002	18
*Bone surface*	0.01		
Calcaneal tuberosity		0.049	13, 19
*Skin*	0.01		
Superior extensor Retinaculum		0.009	8
Calcaneal tendon		0.026	10
*Remainder*	0.12		
Lymphatic nodes		0.010	10, 14, 15
Muscle		0.009	2, 3, 5, 20

### Bone marrow

Bone marrow is one of the largest anatomical structures in the body and represents 4% of the total body mass [23]. The bone marrow mass fraction used in this study was based on the results of two previous studies by Fuller et al. [24] and Les et al. [25]. Fuller et al. measured the bone marrow volume in the thigh and calf area using MRI data. According to their calculations, it was estimated that 100 mm sections of tibia and fibula contained 0.7% of whole body mass bone marrow. Furthermore, Les et al. [25] calculated the bone marrow content of the foot and ankle region using human cadavers and estimated that the structures contained 0.4% of the whole body mass bone marrow. When combining the results of both studies, a total of 1.1% of the bone marrow mass is contained in the ankle area of the examined phantom.

### Bone surface

The bone surface in the ankle region was estimated based on the bone surface-to-volume ratio presented in ICRP 70 [26], and the percentage of total skeletal mass fraction contributed by the exposed bones according to ICRP 89 [27]. The bone surface in the examined region was estimated to represent 4.9% of the full body bone surface.

### Skin

The skin area in the calf and ankle region on layers 23–26 was calculated using the perimeter of each layer multiplied by layer thickness (25 mm). The skin area in foot region layers 27–28 was assessed with a simple planimetric method consisting of placing transparent film on the target and then measuring the area that the film covered. The total body skin area was calculated using the Du Bois-Du Bois formula [28]. The skin of phantom layers 23–28 was estimated to represent 3.5% of the whole body skin area.

### Remainder tissues

Muscles and lymphatic nodes are the only organs in the leg that are included in the remainder tissues defined by ICRP 103 [16].

### Muscles

The total body muscle mass is estimated to weigh 28.000 g [29,30]. The assessment of muscle volume in the ankle and foot was based on a lower extremity property study by Ward et al. [31]. The sum of the muscle fractions contained in the examined volume was estimated to represent 0.9% of total body muscle mass.

### Lymphatic nodes

According to a whole-body lymphoscintigraphy examination performed at the Docrates Cancer Center in Helsinki, Finland, it was estimated that one ankle contains 1% of the lymphatic nodes of the whole body.

The effective dose was obtained from measured organ doses using the guidelines given by the International Commission on Radiological Protection (ICRP 103) [16]. The effective dose E is calculated by the following equation:

2 $$ E={\displaystyle \sum_T{w}_T\cdot {H}_T,} $$

where *w*_*T*_ is the weighting factor of tissue T and H_T_ is the equivalent dose in tissue T. According to the ICRP recommendation, the calculation of effective dose is based on a large number of organs and tissues in the body and the sum of the weighting factors *w*_*T*_ is 1. Muscles and lymphatic nodes are grouped in the calculation as “remainder tissues”. The *w*_*T*_ for the remainder tissues specified by ICRP 103 is 0.12.

The equivalent dose in the bone marrow and the bone surface was calculated by averaging the equivalent doses of the corresponding dosimeters representing the organ. Finally, the contribution to the effective dose was calculated using specific fractions irradiated and weighting factors. The ICRP 103 weighting factors *w*_*T*_ and the fraction of irradiation used in the calculations are shown in Table 3.

## Results

### Siemens sensation open MSCT device

The effective dose attained using the MSCT device was 21.4 μSv. The highest contributor to the effective dose was bone marrow (44%) followed by bone surface (34%), skin (15%), lymphatic nodes (4%) and muscles (3%).

### NewTom 5G CBCT device

The effective dose obtained using the NewTom 5G CBCT device with three different FOVs including the default ankle settings and automatic dose control. Using the 15 × 12 cm FOV with the “standard dose” setting the effective dose was 4.0 μSv. The contributors to the effective dose were as follows: bone surface (30%), bone marrow (44%), skin (19%), lymphatic nodes (5%) and muscles (1%). Using the 18 × 16 cm FOV with the “standard dose” setting the effective dose was 1.9 μSv. The contributors to the effective dose were as follows: bone surface (32%), bone marrow (43%), skin (17%), lymphatic nodes (5%) and muscles (3%). Using the 12 × 8 cm FOV with the “High-Res” setting the effective dose was 14.3 μSv. The contributors to the effective dose were as follows: bone surface (51%), bone marrow (27%), skin (16%), lymphatic nodes (5%) and muscles (1%).

### Planmed verity CBCT device

The effective dose attained on the Planmed Verity CBCT device using the default “medium” ankle settings was 6.0 μSv. The major contributors to the effective dose were bone marrow (45%), bone surface (33%), skin (15%), lymphatic nodes (6%) and muscles (1%).

### Shimadzu FH-21 HR conventional radiography device

The effective doses attained using conventional radiography were 0.5 μSv in the anterior-posterior (AP) position and 1.0 μSv in the lateral position (LAT). Most standard diagnostic procedures comprise both AP and LAT exposures. The sum dose was 1.5 μSv and the bone marrow was the highest contributor (49%) followed by bone surface (23%), skin (18%), lymphatic nodes (6%) and muscles (4%).

**Figure 4:**
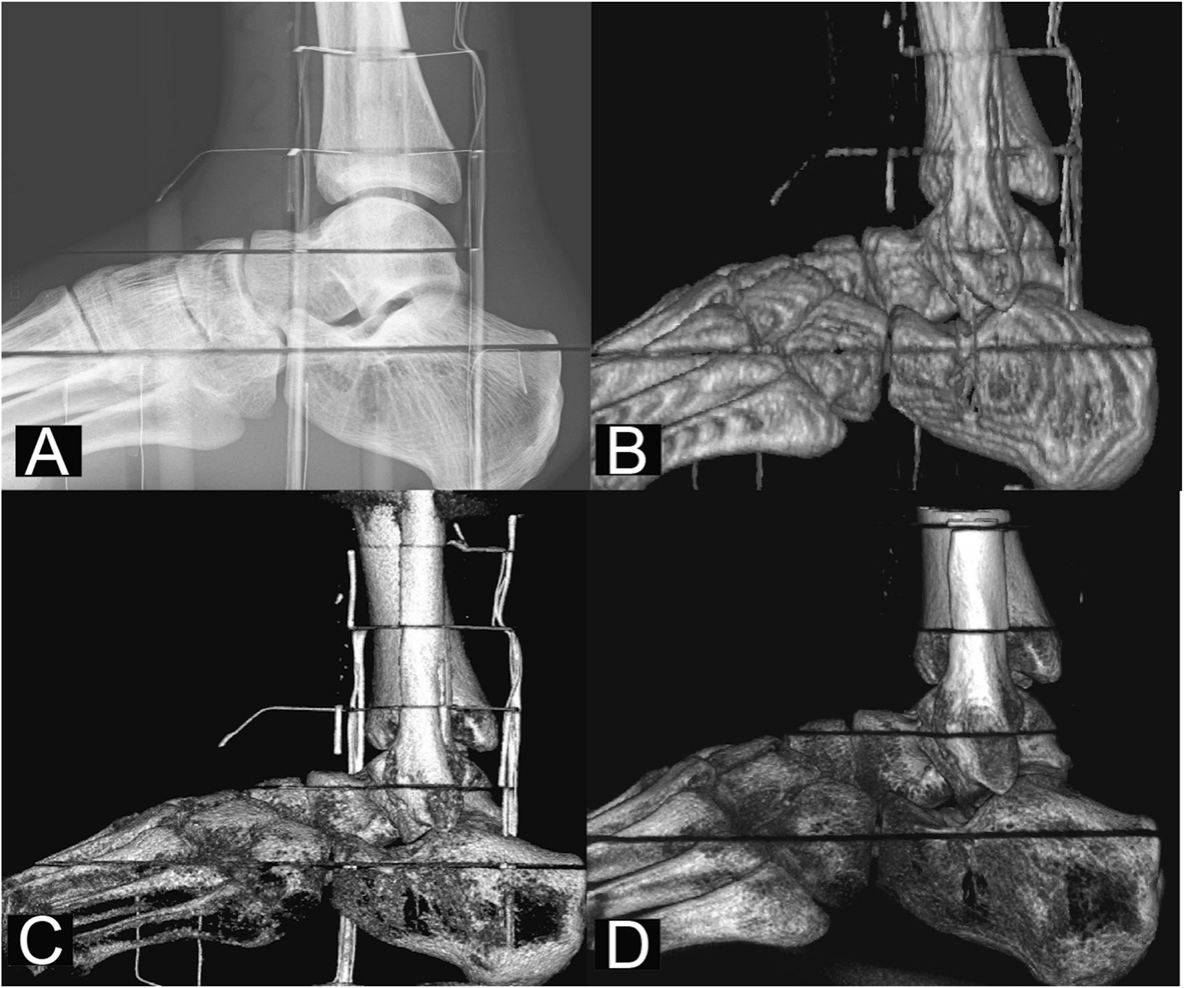
**Sagittal ankle projections using the Shimadzu FH21-HR radiography device (A), the Siemens Sensation Open MSCT device (B), the NewTom 5G CBCT device (18 × 16 cm FOV) (C) and the Planmed Verity CBCT device (D).**

The effective doses and dose contribution of each organ are shown in Table 4. The sagittal projections (cropped images) of the exposed volume using conventional radiography, two CBCT and MSCT devices excluding toes are presented in Figure 4.

**Table 4:** **Equivalent dose contributions and effective doses (μSv) attained in the ankle region using MSCT, CBCT and radiography devices**

	**Siemens**	**NewTom 5G**				**Shimadzu**
	**Sensation**	**CBCT**			**Planmed**	**FH-21 HR**
	**Open**	**Hi Res**	**Standard**	**Standard**	**Verity**	**X-ray**
	**MSCT**	**“12 × 8”**	**“15 × 12”**	**“18 × 16”**	**CBCT**	**AP+LAT**
**Bone marrow**	9.5	3.9	1.8	0.8	2.7	0.7
Bone surface	7.2	7.3	1.2	0.6	1.9	0.3
Skin	3.1	2.2	0.7	0.3	0.9	0.3
**Remainder**						
Lymphatic nodes	0.9	0.8	0.2	0.1	0.3	0.1
Muscles	0.6	0.1	0.1	0.1	0.1	0.1
Effective dose	21.4	14.3	4.0	1.9	6.0	1.5

### Organ and effective dose uncertainty

The type A standard (1SD) uncertainties of the absorbed organ doses were evaluated according to a previous study [15]. The calculated statistical dosimeter uncertainties were as follows (Table 5): Siemens Sensation Open (3-5%), NewTom 5G: “12 × 8 cm” FOV (4-33%) (Table 5), NewTom 5G: “15 × 12 cm” FOV (7-17%), NewTom 5G: “18 × 16 cm” FOV (12-23%), Planmed Verity (5-34%) and Shimadzu FH-21 HR (9-26%). The uncertainties of the point dose measurements were evaluated as the weighted sum of variances and included the statistical measurement error according to a previous investigation [32], uncertainties from the phantom and dosimeter positioning (10%, 10%), x- ray source variation (5%) [19] and cable irradiation uncertainties (1%) [33]. Regardless of the individual point dose variations (Table 5) and the other sources of uncertainties the overall impact of one dosimeter on the effective dose was negligible. The combined uncertainties (u_c_) for a single dosimeter were as follows: Siemens Sensation Open (15-16%), NewTom 5G: “12 × 8 cm” FOV (16-36%), NewTom 5G: “15 × 12 cm” FOV (17-23%), NewTom 5G: “18 × 16 cm” FOV (12-23%), Planmed Verity (16-37%) and Shimadzu FH-21 HR (17-30%). The tissue dose uncertainty depends on the dosimeter uncertainty and the assessed uncertainty of the irradiated tissue fraction *f*_*i*_ (25%). The tissue dose uncertainties attained using e.g. the NewTom 5G CBCT device (12 × 8 cm FOV) was as follows: bone marrow (14%), bone surface (29%), skin (26%) and remainder tissues (19%).

**Table 5:** **Average and (1SD) variation based on six absorbed dose meausurements and combined point dose uncertainty for twenty dosimeters with NewTom 5G (12 × 8 cm FOV)**

***Dosimeter location***	***Dosimeter***	***Measured***	***Dose***	***Point dose***
***in phantom***	***no.***	***dose (mGy)***	***uncertainty (%)***	***uncertainty (%)***
Tibia	1	0.25	33%	36 %
Peroneus brevis	2	0.33	27%	31 %
Gastrocnemius	3	0.52	20%	25 %
Tibia	4	0.48	22%	26 %
Tibialis anterior	5	0.53	20%	25 %
Fibula	6	0.73	17%	22 %
Tibia	7	0.81	16%	22 %
Superior extensor Retinaculum	8	3.86	7%	16 %
Tibia	9	4.04	7%	16 %
Calcaneal tendon	10	7.11	5%	16 %
Fibula	11	6.53	5%	16 %
Lymp.vein	12	10.4	4%	16 %
Posterior calcaneal tuberosity	13	7.71	5%	16 %
Navicular	14	4.09	7%	16 %
Talus	15	4.66	6%	16 %
Metatarsals	16	4.25	6%	16 %
Lymp.vein	17	6.15	5%	16 %
Calcaneus	18	6.45	5%	16 %
Calcaneal tuberosity	19	7.33	5%	16 %
Flexor digitorum brevis	20	3.40	7%	17 %

The expanded (2SD) combined effective dose uncertainties (U_c_) were calculated as a weighted sum of variances of bone marrow, bone surface, and skin and remainder tissue doses according to a previous study [17]. This resulted in 23% for the Siemens Sensation Open MSCT device, 32% for the NewTom 5G device, 23% for the Planmed Verity device and 21% for the Shimadzu FH-21 HR device.

## Discussion

In this study, the organ and effective doses in the ankle area were evaluated on one MSCT device, two CBCT devices and one radiographic device. The organ dose measurements were performed using an anthropomorphic ankle phantom and twenty MOSFET dosimeters positioned in the most radiosensitive areas. The effective doses of the NewTom 5G and Planmed Verity ankle imaging CBCT modalities have not previously been presented.

The highest effective dose (21.4 μSv) was attained using the Siemens Sensation Open MSCT device. The dose was 14 times higher than the dose attained using the conventional device. The effective dose attained using the NewTom 5G CBCT device was 4.0 μSv using the “Standard dose” setting and 15 × 12 cm FOV. This resulted in 1.7 times higher dose than the conventional device. Furthermore the 16 × 18 cm FOV using the “Standard Dose” setting was 1.9 μSv hence 30% higher than the dose attained using the conventional x-ray device. The effective dose of the NewTom 5G CBCT device using the “High-Res” mode and 12 × 8 cm FOV (14.3 μSv) was the 9.6 times the dose of a conventional device but still 33% lower when compared to the MSCT. The dose for Planmed Verity (6.0 μSv) was four times higher than that of conventional radiography, but less than one third of the MSCT dose”.

The CBCT devices evaluated in this study demonstrated significantly lower effective doses when compared with the MSCT device. However, even lower doses were acquired using the conventional 2D radiography device. Interestingly, the two CBCT devices in this study differed in kV and FOV settings. The Planmed Verity CBCT device provides only one (13 × 16 cm) FOV. The effective doses were measured for Planmed Verity retrospectively using lower kVps with the default 48 mAs setting, resulting as follows: 3.9 μSv (80 kVp), 4.8 μSv (84 kVp), 5.3 μSv (88 kVp). The NewTom 5G device did not offer possibilities to change the tube voltage, and it is fitted with a “Safe Beam” automatic exposure control that adjusts the tube current (mA) based on scout images according to factory preset dose levels. The NewTom 5G device, however, allows the user to select from three different fields of view (12 × 8 cm, 15 × 12 cm and 18 × 16 cm) that were also investigated. The “Safe Beam” setting resulted in a much higher dose (14.3 μSv) than those attained on the Planmed Verity device even though a much smaller (12 × 8 cm) FOV with “HiRes” option was used. However, when larger FOVs with “Standard” option were used on the NewTom 5G device, in the effective doses were registered (110 kVp): 4.0 μSv for 15 cm × 12 cm FOV (5.3 mAs) and 1.9 μSv for 18 cm × 16 cm FOV (2.3 mAs). The reduction in the effective dose using larger FOVs could have been caused by the use of the “Standard” option that reduces the mAs. Furthermore, when using the larger FOVs the scout images were larger than the phantom, resulting in an underestimation of the patient attenuation thus yielding to smaller mAs. Presumably this does lead to a reduction in image quality, but it may still provide adequate diagnostic quality.

In a recent study, Ludlow et al. evaluated the effective doses in the foot and ankle area using a conventional radiographic (Siemens) device, a 3D MDCT (Siemens Definition) device and a CBCT (PedCAT) device [7]. The resulting effective doses were 0.6 μSv for the radiographic device, 23 μSv for the MSCT device (120 kVp, 100 mAs) and 1.4 μSv for the CBCT device (100 kVp, 4.5 mAs). The differences in the results compared to those of this study could have been caused by a number of potential differences between the study designs. These include the different field of view, manufactures’ X-ray spectra, exposure parameters, mAs’s, and the bone surface, skin and muscle organ compositions used by Ludlow et al. The major difference between our studies is that the lymphatic nodes and bone marrow structures were included in our study, as proposed by Fuller and Les et al., while they were not included in the study by Ludlow et al. since their bone marrow content was based on the old model by Cristy et al. [34]. According to Cristy et al., there is no active bone marrow in the lower adult extremities. According to Fuller et al. [24], however, 10 cm of the calf section (tibia, fibula) contains 20 cm^3^ of bone marrow. According to McGlamry et al. [35], the proximal tibia has been the most frequently used location to extract bone marrow in volumes between 30–40 cc. Moreover, Les et al. [25] extracted up to 6 cc bone marrow from the calcaneus and, according to Schweinberger et al. [36], the calcaneus and proximal tibial metaphysis offer a significant amount of bone marrow. In contrast to Ludlow’s study, our study included the bone marrow, which was the major contributor to the effective dose. When the bone marrow contributions were extracted from our results and the mAs’ were scaled to match those of Ludlow et al. the differences between the two studies were less than 20%. The differences were small considering the variations in FOVs, X-ray spectra, exposure parameters, phantom tissue composition and measurement methods.

Earlier, Huda et al. [37] assessed the effective dose in the ankle region using conventional radiography (70 kVp, 10 mAs). They observed a 0.72 μSv upper limit of the effective dose. The differences in the doses were caused by the number of projections taken. Namely, in this study AP+LAT projections were taken while in the study by Huda et al. only one exposure was performed. Furthermore, in a study by Cross et al. [38], the effective dose and radiation risk associated with ionizing radiation in sports medicine were investigated. According to their findings, the effective dose for common radiographic procedures in the ankle and foot area resulted in a higher (4 μSv) dose than that attained in this study.

In a previous study, Biswas et al. investigated the radiation exposure from musculoskeletal computed tomography scans. In their study, the imaging parameters were recorded and calculator software was used to attain the effective doses according to a protocol derived from publication SR250 of the National Radiological Protection Board of the United Kingdom [39]. Tube voltage and tube current (mean ± SD) were 120 kVp and 143 ± 91 mA (mean ± SD), respectively. According to their study, the effective dose resulting from the ankle scan was 70 μSv. Our effective dose result was one third of that obtained by Biswas et al.

All effective doses were evaluated in this study based on manufacturer recommendations and, therefore, the results of this study should be interpreted with caution. A further limitation of this study is the lack of image quality assessment that is essential when choosing the appropriate exposure parameters. Another setback of this study is the lack of clinical evaluation of the different imaging modalities. Nevertheless all CBCT volumes in this study were reconstructed with smaller voxel sizes than those used on the MDCT device. The smaller voxel size can be an advantage in the detection of non-displaced fractures in clinical settings. On the other hand, CBCT devices have a higher noise and lower contrast-to-noise ratio than MDCT devices. This might be a disadvantage in the delineation of fractures. Nevertheless in such cases the use of a MDCT device could be advantageous despite of the higher effective dose induced.

A further drawback of this study was the lack information on the quantification of the proportions of red and yellow bone marrow in the ankle region; this study assumes 100% red bone marrow and no yellow bone marrow, possibly leading to an overestimation of the effective dose. Another source of error in this study could be the use of two projections instead of the three projections recommended by ACR [40] in conventional radiography (AP, LAT, (mortise) oblique projections).

The setback of MOSFET dosimeters is that they are not tissue equivalent and become visible on the radiographs. In this study the exposures were, however, taken only to evaluate the effective dose and thus the visibility of the MOSFET dosimeters in the images were not considered problematic.

## Conclusions

Compared with the conventional 2D radiographic device, this study showed a 14-fold effective dose for a standard MSCT protocol and between a 1.3-4 fold effective dose for standard CBCT protocols The results of this study showed a large variability in the effective dose values attained on the CBCT devices using different scan modes and FOVs. Furthermore, when compared with MSCT devices, the two CBCT devices assessed in this study offer a promising low-dose, three-dimensional alternative for ankle imaging.
